# Development of a High-Temperature-Resistant Encapsulated Gel Breaker for Polymer Gels and Evaluation of Its Performance

**DOI:** 10.3390/gels12060479

**Published:** 2026-05-29

**Authors:** Chenghao Zhang, Jingbin Yang, Zhongyi Wang, Mengyao Wang, Yuan Liu

**Affiliations:** 1 Zhejiang University, Hangzhou 310027, China; 3200102159@zju.edu.cn; 2 CNPC Engineering Technology Research Institute Co., Ltd., Beijing 102206, China; wangmydr@cnpc.com.cn; 3 School of Petroleum Engineering, China University of Petroleum (East China), Qingdao 266580, China; kk18252127631@163.com; 4 Drilling Fluid Technology Service Center of Sinopec Shengli Petroleum Engineering Co., Ltd., Dongying 257000, China; wzy0512xx@126.com

**Keywords:** lost circulation, encapsulated gel breaker, high-temperature resistance, temporary plugging

## Abstract

To address the poor temperature resistance of conventional gel breakers, the uncontrollable gel-breaking time, and the risk of secondary reservoir damage during temporary plugging of fractured formations with polymer gels, a high-temperature-resistant double-shell encapsulated gel breaker, UF-EC/SA, was prepared using oil-phase phase separation combined with in situ polymerization. In this material, urea-formaldehyde resin (UF) served as the outer shell, ethyl cellulose (EC) as the inner shell, and sulfamic acid (SA) as the core. Unlike conventional single-shell persulfate or directly added acid breakers, this double shell design integrates a thermally resistant UF barrier, a diffusion-controlling EC layer, and an acid core to delay premature gel degradation while enabling subsequent cleanup. The physical structure and sustained-release behavior of the capsules were characterized by scanning electron microscopy (SEM), Fourier transform infrared spectroscopy (FTIR), thermogravimetric analysis (TGA), powder X-ray diffraction (XRD), and conductivity measurements. The compatibility between the encapsulated breaker and the polymer gel, as well as the effects of salinity and breaker dosage on the rheological properties of the gel, were investigated. The regulatory effects of temperature and capsule dosage on gel-breaking performance were studied in detail. In addition, high-temperature/high-pressure displacement experiments were conducted to evaluate the temporary plugging performance of the gel containing the encapsulated breaker in fractured cores and packed-sand tubes. The results showed that the prepared capsules had good sphericity and a dense shell structure, with an encapsulation efficiency of 76.7%. The capsules exhibited temperature resistance up to 150 °C and favorable sustained-release characteristics. The UF-EC/SA breaker showed good compatibility with the polymer gel and did not inhibit gelation within the temperature range of 80–150 °C or at dosages of 0–16 wt.%. The gel maintained good mechanical strength even in highly mineralized brines. At 150 °C and a capsule dosage of 16 wt.%, the gel was completely broken within 2.5 d; the residue concentration was only 351 mg/L, and the residue size was mainly distributed within 100–500 μm. The high-temperature/high-pressure displacement tests demonstrated that the gel containing 16 wt.% capsules achieved a maximum breakthrough pressure of 5.16 MPa in a 3 mm wedge-shaped fracture core, and the pressure remained stable for 5 d. After gel breaking, the residue could be readily flowed back, indicating excellent synergy between temporary plugging and subsequent gel breaking. Therefore, the UF-EC/SA encapsulated breaker provides a new technical option for efficient gel breaking in high-temperature fractured formations.

## 1. Introduction

Severe lost circulation in fractured formations is one of the most difficult downhole complications to control and remains a global technical challenge in oil and gas exploration and development. Under formation pressure, drilling fluids can flow into fractured formations through loss channels, causing large volumes of drilling-fluid loss and excessive consumption of plugging materials. This not only may induce blowouts, wellbore collapse, and pipe sticking but can also lead directly to abandonment of the wellbore and major economic losses, thereby seriously hindering oil and gas development [1,2,3,4]. During temporary plugging operations, different oilfields adopt different plugging technologies depending on the block conditions and loss intervals. Among them, polymer-gel temporary plugging technology has been widely applied. Conventional gel plugging materials are not strictly limited by the geometry of the loss channel and can therefore effectively treat lost circulation in fractured reservoirs. However, in practical applications, polymer gels often suffer from poor post-gel degradation, uncontrollable gel-breaking time, and secondary formation damage [5,6]. A common solution is to add a breaker during temporary plugging so that the gel can later be degraded, but the breaker performance is strongly influenced by the formation environment, especially temperature. Under high-temperature conditions, conventional breakers may cause the gel to lose viscosity too early, resulting in poor controllability of the temporary plugging effect and an adverse impact on field operations [7,8].

To maintain gel viscosity during the plugging stage while minimizing potential damage to oil and gas reservoirs, several types of gel breakers have been developed, including oxidative breakers, enzymatic breakers, acid breakers, and encapsulated breakers [9,10,11,12]. Oxidative breakers are effective for polymer-chain scission but their activity is highly sensitive to temperature and may cause premature viscosity loss under high-temperature conditions. Enzymatic breakers generally show better selectivity, but their activity is restricted by temperature and pH. Directly added acid breakers can rapidly destroy cross-linking nodes, but early acid–gel contact may inhibit gelation and increase corrosion risk. Encapsulated breakers reduce these drawbacks by isolating the active breaker from the gel and releasing it gradually through a polymeric shell [13,14]. However, most reported systems are single-shell, persulfate-based, or designed mainly for fracturing fluids rather than high-strength temporary plugging gels in large fractures. For example, Lo et al. [15] prepared an encapsulated ammonium persulfate breaker that released after 6 h at 150 °F. Al-Hulail et al. [16] developed an encapsulated breaker with ammonium persulfate as the core material, which could withstand temperatures up to 330 °F and provided a delayed gel-breaking time of 170 min in a carboxymethyl-carboxypropyl guar fracturing system. Waters and DeLeon [17] demonstrated field applications in the Red Fork Formation. These studies confirm the value of delayed breakers, but they do not fully address the simultaneous requirements of high-temperature storage stability, controllable acid release, compatibility with temporary plugging gels, and post-breaking flowback in fractured formations.

Polymer composite gels used in temporary plugging exhibit the advantages of environmental friendliness, low toxicity, good structural integrity, and controllable size [18]. Under relatively harsh service conditions, swellable polymer gels can migrate into deeper formation zones and substantially improve formation heterogeneity, thereby enhancing oil recovery [19,20]. Therefore, polymer composite gel systems are particularly suitable for lost-circulation control in reservoirs with poor geological conditions and low permeability [21]. Nevertheless, these systems also present practical limitations. Urdahl et al. [22] reported a temporary gel plug that was convenient for field preparation and could flow back after gel breaking, but the gel-breaking process remained dependent on the operating environment. Cole [23] and Evans [24] developed environmentally friendly hydroxyethyl-cellulose-based fluid-loss control systems with low-toxicity crosslinkers; however, their use relies on specific crosslinking and pH-control conditions. Chang et al. [25] developed a crosslinked HEC system for high-permeability and high-temperature reservoirs up to 143 °C, but subsequent cleanup still required chemical removal. Khater et al. [26] demonstrated a low-damage gel plug for openhole completion, yet the flowback effect de-pended on the efficiency of the acid breaker. Mebratu et al. [27] reported a high-strength long-life polymer gel barrier with good salinity tolerance, but long-term stability can also prolong cleanup. Wilson [28] and Halliburton [29] reported solids-free fluid-loss pills for high-temperature reservoirs; these systems effectively reduced fluid loss but required chemical removal after the operation. Thus, the key challenge is not only to form a strong gel plug but also to achieve delayed, controllable, and low-residue gel breaking after the plugging period.

Accordingly, the research gap addressed in this work is the lack of a high-temperature-resistant encapsulated acid breaker that can remain compatible with polymer temporary plugging gels during injection and gelation but release the breaker after plugging to achieve low-residue cleanup. The originality of this work lies in the construction of a UF-EC/SA double-shell structure: the UF outer shell improves thermal and mechanical stability, the EC inner shell provides a secondary diffusion barrier, and the sulfamic-acid core supplies an acid source for destroying the gel network after release. The capsules were prepared by combining oil-phase phase separation with in situ polymerization, and their structure, thermal stability, compatibility, salt tolerance, gel-breaking controllability, residue characteristics, and temporary plugging/flowback performance were systematically evaluated. A dual release mechanism involving temperature-assisted permeation and pressure-triggered shell rupture was further proposed. Ultimately, a temporary plugging and cleanup strategy was established for fractured formations at 150 °C, featuring stable plugging, controllable gel breaking, low residue concentration, and easy flowback.

## 2. Results and Discussion

### 2.1. Encapsulation Efficiency Analysis

#### 2.1.1. Encapsulation Efficiency of EC/SA Capsules

As shown in Table 1, decreasing the core-to-wall mass ratio from 10:1 to 10:3 increased the EC/SA encapsulation efficiency from 63.4% to 85.3%, indicating that a higher EC fraction promoted more complete deposition of the wall material around the sulfamic acid core. When the core-to-wall ratio was further reduced to 10:4, the encapsulation efficiency increased only slightly from 85.3% to 85.6%. This 0.3 percentage-point improvement was not sufficient to justify the additional EC consumption, and slight capsule adhesion was also observed at the higher wall-material dosage. Therefore, the 10:3 ratio was selected as the optimal balance between encapsulation efficiency, product dispersibility, and preparation cost.

#### 2.1.2. Encapsulation Efficiency of UF-EC/SA Capsules

As shown in Table 2, the encapsulation efficiency of the UF-EC/SA capsules first increased and then decreased with increasing UF prepolymer/EC-SA capsule mass ratio. When the ratio was increased from 10:1 to 10:5, the encapsulation efficiency rose from 72.8% to 76.7%, showing that sufficient UF resin was necessary to form a continuous outer shell on the EC/SA capsules. Further increasing the ratio to 10:7 or 10:9 reduced the encapsulation efficiency to 70.7% and 61.2%, respectively. This decrease may be attributed to excessive UF resin increasing the viscosity of the dispersed phase and promoting capsule adhesion or incomplete separation during washing. Therefore, a mass ratio of 10:5 was selected because it produced the highest encapsulation efficiency while avoiding unnecessary shell-material consumption.

### 2.2. Properties of the UF-EC/SA Double-Shell Encapsulated Breaker

#### 2.2.1. FTIR Analysis

**Figure 1:**
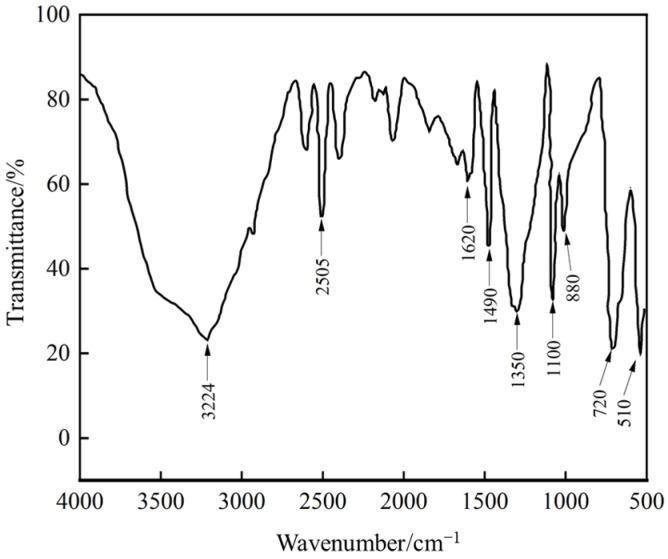
FTIR spectrum of the UF-EC/SA encapsulated breaker.

As shown in Figure 1, several characteristic absorption peaks appeared at specific wavenumbers. The peaks at 1490, 1350, 720, and 510 cm^−1^ were assigned to the characteristic absorptions of sulfonic groups, indicating the presence of sulfamic acid functionalities in the sample. The absorption at 880 cm^−1^ corresponded to the characteristic peak of ethyl groups, demonstrating the presence of ethyl-containing structures. The peak at 1100 cm^−1^ was attributed to the intramolecular ether bond of cellulose. In addition, a broad absorption at 3224 cm^−1^ resulted from the overlap of N-H and O-H stretching vibrations. A relatively broad peak at 2505 cm^−1^ originated from the C=O stretching vibration of amide bonds, further confirming the presence of amide structures. Meanwhile, the -OH absorption around 1500 cm^−1^ became weaker, whereas a new absorption at 1620 cm^−1^ corresponding to the C-N and N-H stretching vibrations in amide bonds appeared. These results indicate that the prepared encapsulated breaker simultaneously contained UF resin, EC, and sulfamic acid.

#### 2.2.2. Thermogravimetric Analysis

**Figure 2:**
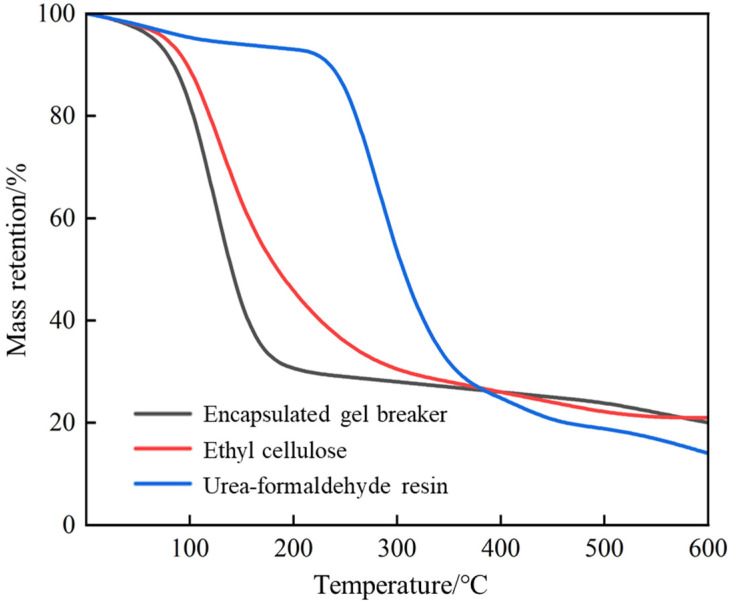
Thermogravimetric analysis of the UF-EC/SA encapsulated breaker and shell materials.

Thermogravimetric analysis is an important method for evaluating material stability. According to the TG curve of the encapsulated breaker in Figure 2, the mass loss in the range of 0–100 °C was relatively small, which can be attributed to the evaporation of water, free formaldehyde, and other small molecules on the capsule surface as the temperature increased. In the range of 120–140 °C, the mass loss increased rapidly because the shell ruptured and decomposed, leading to rapid release of the core material. In the range of 200–600 °C, the mass loss decreased more slowly as the shell further decomposed. The TG curve of EC showed that the temperature resistance of EC was lower than that of UF resin and that EC underwent rapid mass loss in the range of 100–200 °C. In contrast, the TG curve of UF resin showed slow mass loss in the range of 0–300 °C, with the highest mass-loss rate occurring at 310–350 °C, where UF resin decomposed rapidly. Further decomposition continued in the range of 400–600 °C, during which the nitrogen-containing residues in the resin were gradually reduced. These results demonstrate that both the UF prepolymer and EC possess a certain degree of high-temperature resistance and can be safely stored at room temperature, while the capsules can be activated for gel-breaking operations under formation temperatures of 120–140 °C. Comparison of the three TG curves further suggests that increasing the shell thickness could improve the temperature resistance of the encapsulated breaker and may provide a route to achieve more precise control of gel-breaking time according to specific operational requirements.

#### 2.2.3. Micromorphology Analysis

**Figure 3:**
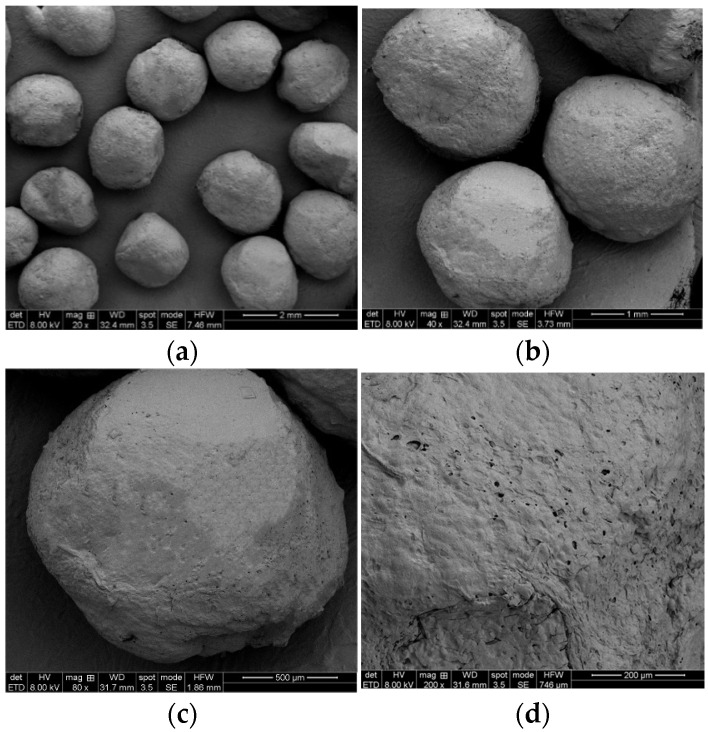
SEM images of the encapsulated breaker at different magnifications: (**a**) 20×; (**b**) 40×; (**c**) 80×; (**d**) 200×.

Based on the preparation process of the high-temperature-resistant encapsulated breaker, the obtained UF-EC/SA capsules were observed by SEM, as shown in Figure 3. Figure 3a,b shows that the optimized UF-EC/SA capsules were nearly spherical and had a uniform particle size of approximately 1.5 mm. Upon local magnification, Figure 3c reveals that the surface of individual particles was relatively smooth and exhibited no obvious shell rupture or collapse. At higher magnification (Figure 3d), the capsule surface displayed a honeycomb-like porous morphology with a non-uniform pore density. These pores were generated by evaporation of surface moisture during the final drying step. In addition, when the capsules dried at 60 °C were removed from the oven, thermal expansion and contraction also contributed to the formation of wrinkles and pores.

#### 2.2.4. Powder X-Ray Diffraction Analysis

**Figure 4:**
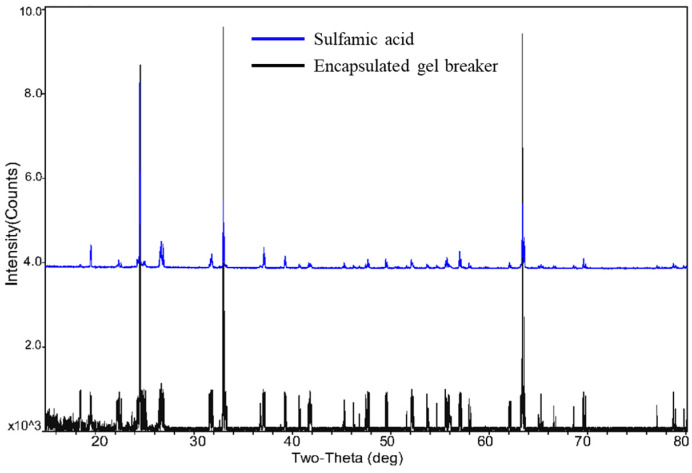
XRD patterns of sulfamic acid and the UF-EC/SA encapsulated breaker.

To determine whether the physicochemical properties of sulfamic acid changed after encapsulation and whether the UF resin and EC successfully coated the sulfamic acid, XRD analysis was performed on both pure sulfamic acid and the encapsulated breaker. As shown in Figure 4, the major diffraction peaks of the encapsulated breaker were located at the same positions as those of sulfamic acid, and the sharp peaks of the two patterns overlapped well. Because EC and UF resin are essentially amorphous, no obvious diffraction peaks attributable to these shell materials were observed. These results indicate that the crystal structure of sulfamic acid remained intact after double encapsulation with EC and UF resin, and its physicochemical properties were not altered, thereby confirming the feasibility of the preparation process.

### 2.3. Compatibility and Gel-Breaking Performance of the UF-EC/SA Double-Shell Encapsulated Breaker in Polymer Gels

#### 2.3.1. Salt Tolerance of the UF-EC/SA Double-Shell Encapsulated Breaker in the Polymer Gel System

Effect of monovalent salt ions on gelation performance. With the increasingly complex geological conditions encountered during oilfield drilling, the salinity of formation water has become progressively higher. In field applications, formation water is often used directly to prepare gel fluids. Therefore, it is necessary to investigate the effect of water salinity on gelation performance. To ensure that the gel strength after adding UF-EC/SA capsules could still meet construction requirements, a capsule dosage of 8 wt.% was selected based on the rheological evaluation in the previous section.

**Figure 5:**
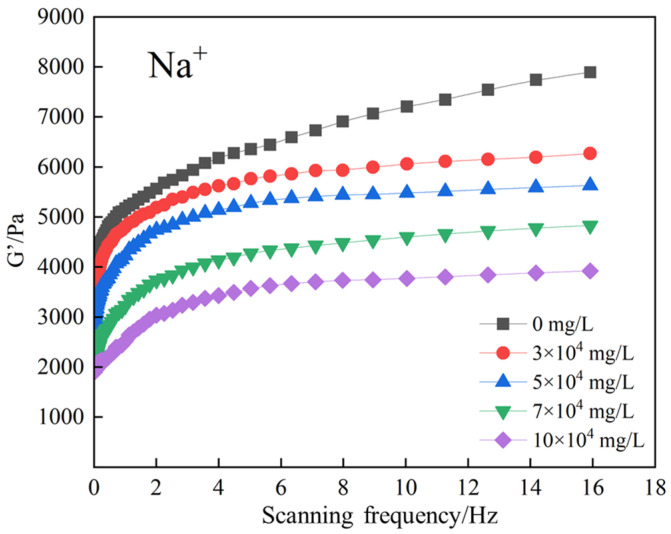
Effect of monovalent ion concentration on the storage modulus of the gel.

As shown in Figure 5, after adding 8 wt.% UF-EC/SA capsules, the storage modulus of the gel increased with increasing sweep frequency under all Na^+^ concentrations, while the increase became less pronounced when the frequency exceeded 2 Hz. As the salinity increased, the storage modulus decreased. At a frequency of 16 Hz, the storage modulus of the gel prepared without monovalent salts reached approximately 8000 Pa, whereas that of the gel prepared in a 100,000 mg/L Na^+^ solution was approximately 3600 Pa, a difference of 4400 Pa. The presence of monovalent salt ions compressed the three-dimensional gel network, weakened its resistance to elastic deformation, and thereby reduced the gel strength.

**Figure 6:**
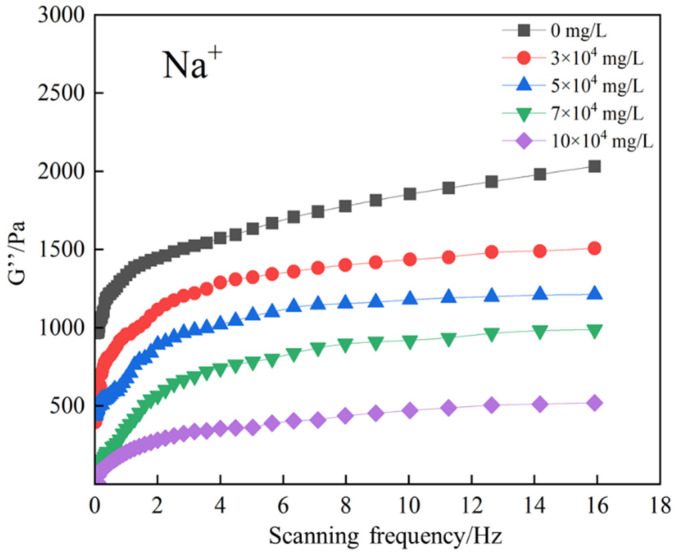
Effect of monovalent ion concentration on the loss modulus of the gel.

As shown in Figure 6, the loss modulus displayed a trend similar to that of the storage modulus. With increasing sweep frequency, the loss modulus gradually increased under all salinity conditions. In the frequency range of 0–2 Hz, the loss modulus increased rapidly for all five groups, whereas above 2 Hz the increase became slower. At 16 Hz, the gel prepared in deionized water had the highest loss modulus, approximately 2000 Pa, while the gel prepared in 100,000 mg/L Na^+^ brine had the lowest value, approximately 500 Pa. As the monovalent salt concentration increased, the loss modulus decreased, indicating that the spatial structure of the gel was significantly affected and that aggregation or clumping may have occurred. Nevertheless, comparison of Figure 5 and Figure 6 shows that, at the same monovalent salt concentration, the storage modulus remained higher than the loss modulus, indicating that the gels still maintained a reasonably good network structure and acceptable post-gel properties even in a monovalent salt environment.

**Figure 7:**
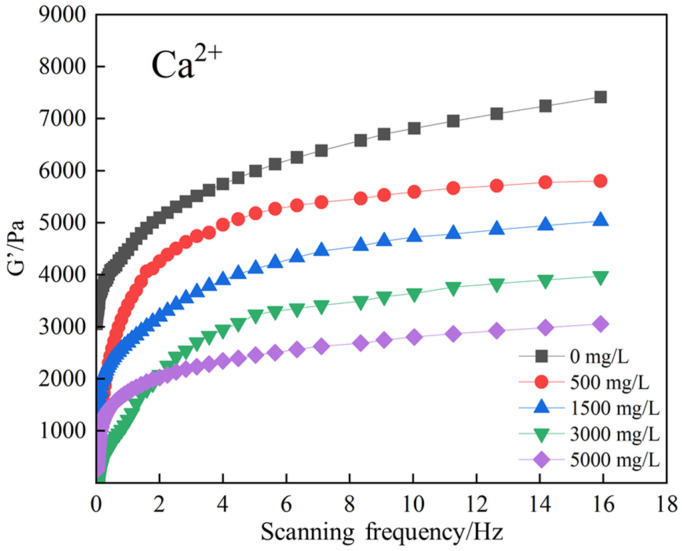
Effect of divalent ion concentration on the storage modulus of the gel.

As shown in Figure 7, the storage modulus increased rapidly in the sweep-frequency range of 0–2 Hz for gels prepared at different Ca^2+^ concentrations, whereas the increase became slower when the frequency exceeded 2 Hz. At 16 Hz, the storage modulus of the gel without divalent salt reached approximately 7800 Pa. When the Ca^2+^ concentration was 1500 mg/L, the storage modulus was about 5000 Pa and, when the Ca^2+^ concentration was 5000 mg/L, it decreased to approximately 3000 Pa, the lowest among the five groups. These results show that the storage modulus decreased with increasing Ca^2+^ concentration because the divalent ions compressed the polymer chains in the gel network and reduced the elasticity of the gel. Nevertheless, a storage modulus of 3000 Pa at 5000 mg/L Ca^2+^ still indicates that the gel containing the encapsulated breaker retained acceptable mechanical strength.

**Figure 8:**
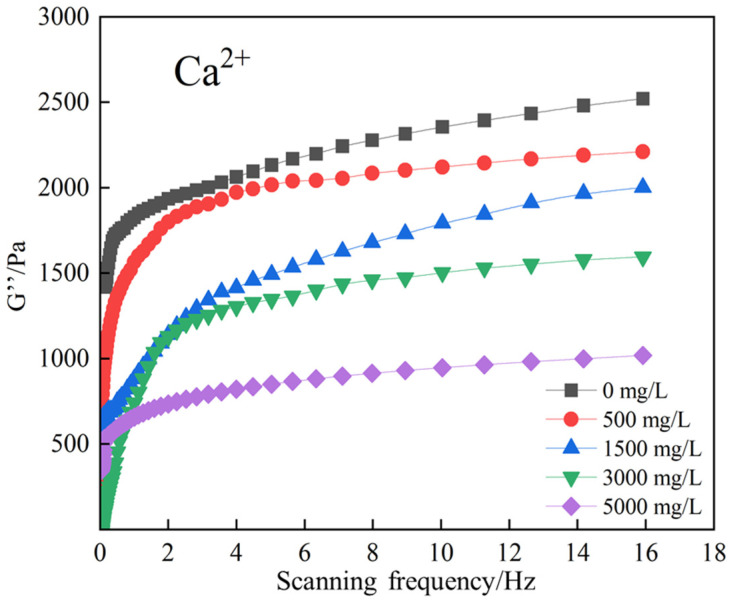
Effect of divalent ion concentration on the loss modulus of the gel.

As shown in Figure 8, the loss modulus in the divalent salt environment increased with frequency in a manner similar to that observed in monovalent salt solutions. In the range of 0–2 Hz, the loss modulus increased rapidly for all groups and then increased more slowly above 2 Hz. At 16 Hz, the loss modulus was approximately 2500 Pa in the absence of Ca^2+^, 2000 Pa at 1500 mg/L Ca^2+^, and 1000 Pa at 5000 mg/L Ca^2+^. As the divalent ion concentration increased, the loss modulus decreased, indicating that the viscous component of the gel weakened and the elastic performance was affected. However, comparison of Figure 7 and Figure 8 shows that, under all Ca^2+^ concentrations, the storage modulus was higher than the loss modulus at the same frequency, indicating that the gels stored more energy elastically and could recover their shape relatively well after the external force was removed.

#### 2.3.2. Effects of Temperature and UF-EC/SA Capsule Dosage on Gel-Breaking Performance

With increasing drilling and production depth, commonly used encapsulated breakers often exhibit excessively fast release and a narrow temperature-adaptation window, which may cause premature viscosity loss of the gel in high-temperature formations. The UF-EC/SA encapsulated breaker prepared in this study exhibits higher thermal resistance and better compatibility with the gel fluid, allowing it to be mixed with the gel solution at the surface and injected downhole together. In this way, the gel strength remains stable during the early stage of the operation, whereas the breaker is released after the operation to degrade the gel and reduce the residue level during flowback. By investigating the effects of capsule dosage and environmental temperature on the gel-breaking performance, the release and breaking mechanism can be elucidated, and the required capsule dosage under different formation temperatures can be determined to achieve controllable gel-breaking time.

**Figure 9:**
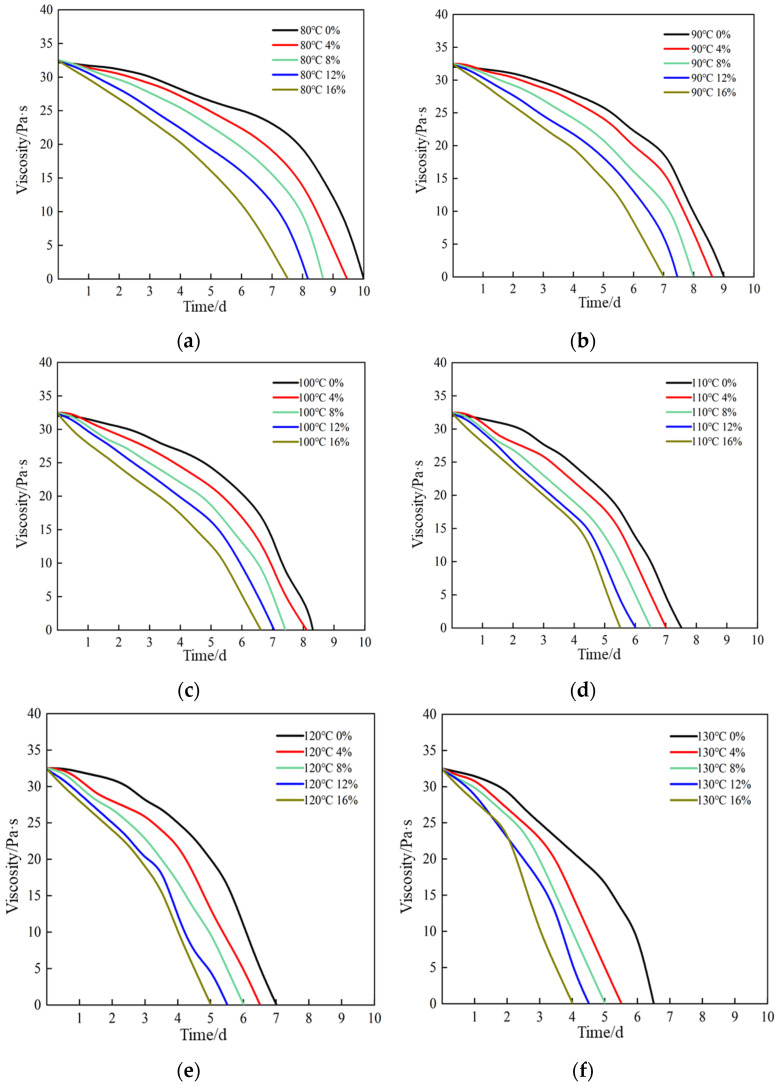

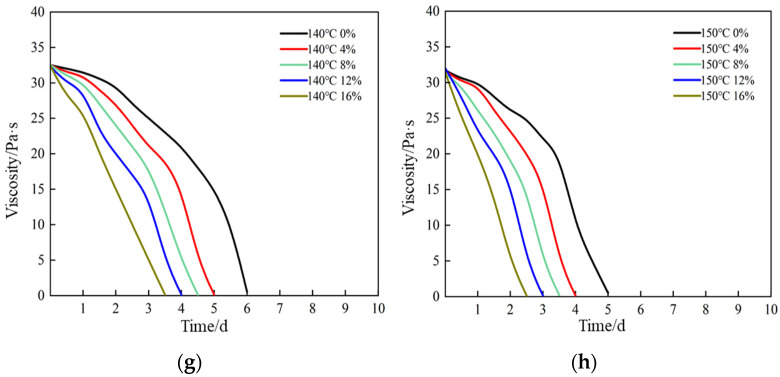
Effects of temperature and capsule dosage on gel viscosity: (**a**) 80 °C; (**b**) 90 °C; (**c**) 100 °C; (**d**) 110 °C; (**e**) 120 °C; (**f**) 130 °C; (**g**) 140 °C; (**h**) 150 °C.

In this section, gel viscosity was used as the criterion to determine whether the gel was broken. According to the petroleum industry standard, a viscosity of 5 mPa s or below was considered complete gel breaking. The experimental temperature range was 80–150 °C and the capsule dosage was 0%, 4%, 8%, 12%, and 16%. As shown in Figure 9a, at 80 °C, the fastest complete gel-breaking time was 7.5 d. As shown in Figure 9b–h, the shortest complete gel-breaking times were 7.0 d at 90 °C, 6.5 d at 100 °C, 5.5 d at 110 °C, 5.0 d at 120 °C, 4.0 d at 130 °C, 3.5 d at 140 °C, and 2.5 d at 150 °C. These results demonstrate that the gel-breaking time decreased as the temperature increased. In addition, at a given temperature, a higher dosage of the encapsulated breaker led to a shorter gel-breaking time. By measuring gel viscosity at different temperatures and capsule dosages, the gel-breaking time under the temperature range of 80–150 °C can be determined, thereby providing a basis for practical design of field operations.

**Figure 10:**
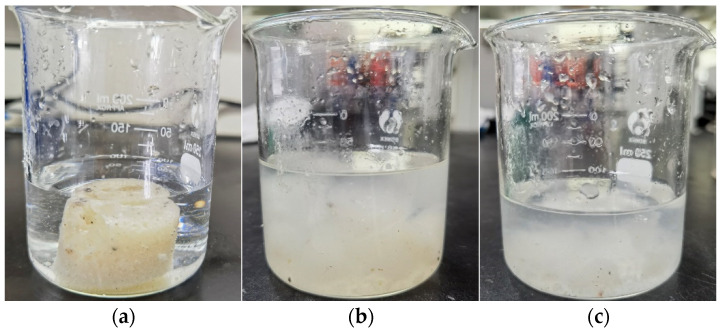
Gel degradation process at 90 °C without the encapsulated breaker: (**a**) Day 1; (**b**) Day 5; (**c**) Day 9.

**Figure 11:**
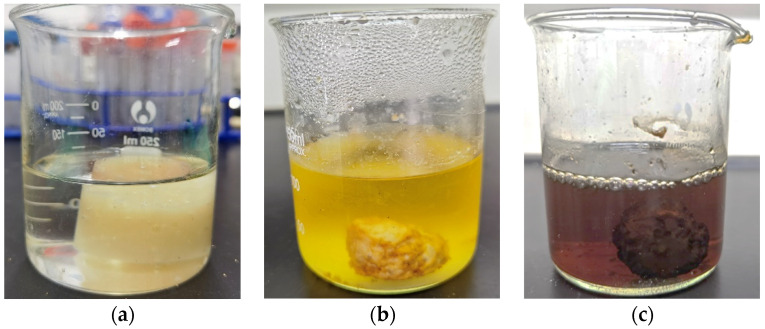

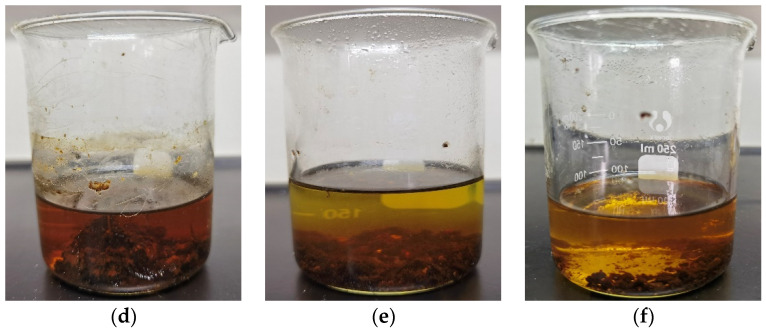
Photographs of the gel-breaking process at 120 °C with 8 wt.% UF-EC/SA capsules: (**a**) Day 1; (**b**) Day 2; (**c**) Day 3; (**d**) Day 4; (**e**) Day 5; (**f**) Day 5.

**Figure 12:**
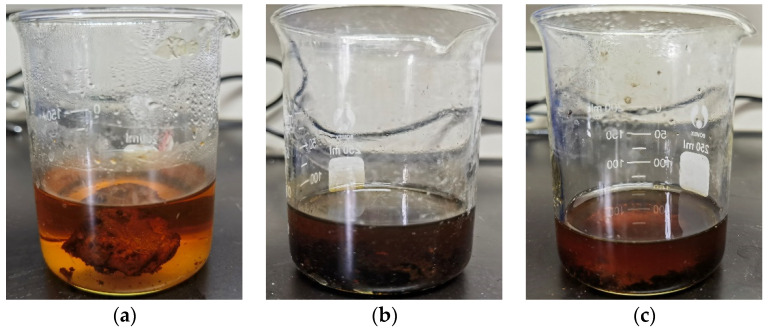
Photographs of the gel-breaking process at 150 °C with 12 wt.% UF-EC/SA capsules: (**a**) Day 1; (**b**) Day 2; (**c**) Day 3.

At 90 °C without the encapsulated breaker, the degradation process of the gel is shown in Figure 10. The gel continuously absorbed water and swelled, and the gel blocks gradually cracked and disintegrated. On Day 9, the gel in the solution appeared as viscous flocculent aggregates. At 120 °C with 8 wt.% capsules, the gel-breaking process is shown in Figure 11. Complete gel breaking required 6 d and, on Day 6, no bulk gel remained in the solution; only solid residues mainly derived from the capsule shells were observed. At 150 °C with 12 wt.% capsules, the gel states at different times are shown in Figure 12. On Day 1, the gel structure remained intact; on Day 2, the gel had disintegrated into small pieces; and, on Day 3, only solid residues remained, indicating complete gel breaking.

#### 2.3.3. Temporary Plugging Performance of the UF-EC/SA Double-Shell Encapsulated Breaker in the Polymer Gel System

To further clarify the temporary plugging performance of the gel after the encapsulated breaker entered the pore/fracture space during operation and to verify whether the gel could maintain effective sealing for a certain period of time, the gel-breaking behavior was tested in steel fracture cores and packed-sand tubes representing different fracture geometries. The temperature was set to 120 °C, the gelation time was 10 h, and the capsule dosage was 16 wt.%; the control group contained no capsules. Using a high-temperature/high-pressure plugging-displacement apparatus, water flooding was conducted after gelation. During pressurization, once the pressure exhibited a fluctuating downward trend, pressurization was stopped and the pressure at that moment was maintained; this value was defined as the maximum breakthrough pressure. The initial breakthrough pressure was defined as the peak pressure at which the pressure curve first began to fluctuate downward during water flooding. The final pressure-release pressure was defined as the pressure immediately before the abrupt pressure drop caused by formation of a flow channel after gel breaking. The pressure-holding time was the time interval between and the abrupt pressure release. These definitions were used consistently for all fracture-core and packed-sand-tube tests. The outlet-end pressure variation for the different media was recorded to determine the gel-breaking and flowback time.

Temporary plugging effect in a parallel fracture with a 5 mm outlet width was assessed. A steel core with a 5 mm outlet parallel fracture was used to simulate the formation fracture width. The gel containing 16 wt.% capsules was injected into this core according to the experimental procedure, and the control group consisted of the gel without capsules. After injection, gel overflow was observed at the outlet ends of both groups, indicating that the inner space of the fracture core was fully filled with the injected gel. After gelation at 120 °C, water flooding was performed and the outlet pressure was recorded.

**Figure 13:**
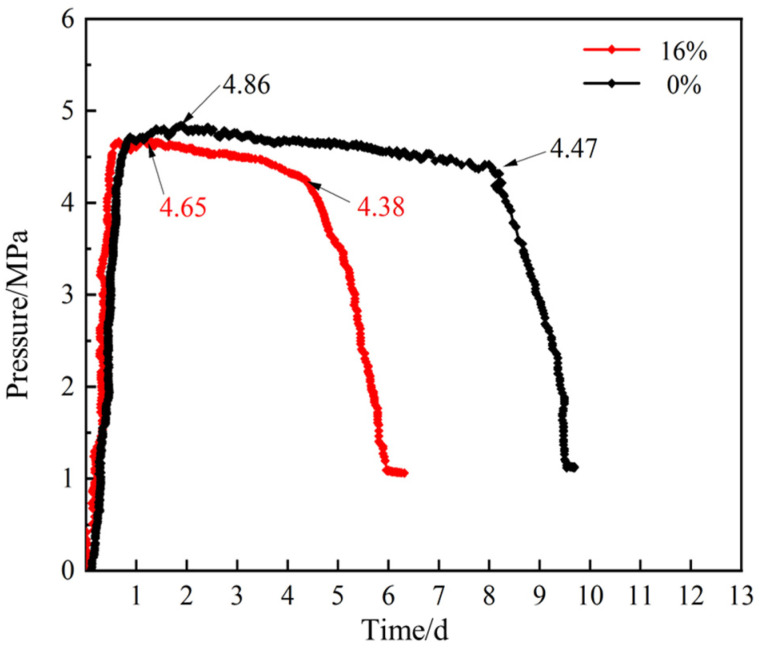
Breakthrough pressure in the parallel fracture core with a 5 mm outlet width.

**Figure 14:**
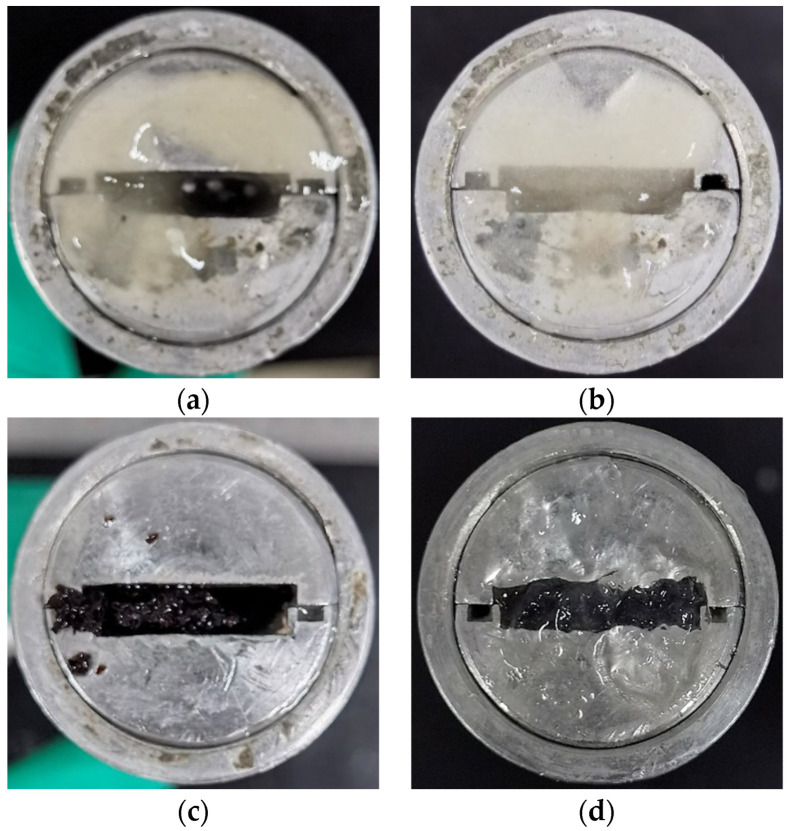
Outlet-end states of the 5 mm parallel fracture core under different conditions: (**a**) with capsule gel breaker; (**b**) without capsule gel breaker; (**c**) after gel breaking with capsule gel breaker; (**d**) after gel breaking without capsule gel breaker.

The pressure-change curves obtained after data processing are shown in Figure 13, and the peak of each curve corresponds to the maximum breakthrough pressure. The maximum breakthrough pressure of the gel containing 16 wt.% capsules was 4.65 MPa. Thereafter, the pressure declined slowly because a small fraction of the capsules released their core material during pressurization, thereby reducing the gel strength. When the pressure dropped instantaneously at 4.38 MPa, the pressure-holding period was 4.5 d. As shown in Figure 14c, gel residues remained at the outlet, indicating that a distinct flow channel had formed in the fracture core and caused rapid pressure release. The maximum breakthrough pressure of the gel without capsules was 4.86 MPa. The pressure was released abruptly at 4.47 MPa, and the pressure-holding period was 8 d. As shown in Figure 14d, the outlet residues were larger gel blocks, which were unfavorable for flowback. These results indicate that, in a fracture with a width of 5 mm, the gel containing the encapsulated breaker could still provide effective temporary plugging for a certain period and, after gel breaking, the residue particle size was smaller than the fracture width, allowing more complete flowback cleanup.

Temporary plugging effect in a wedge-shaped fracture with a 3 mm outlet width. To simulate a wedge-shaped formation fracture with decreasing aperture toward depth, a steel wedge-shaped fracture core with an inlet width of 5 mm and an outlet width of 3 mm was used. The gel containing 16 wt.% capsules was injected according to the same procedure, and the control group contained no capsules. After injection, gel flow was observed at the 3 mm outlet ends of both groups, confirming that the interior of the wedge-shaped core was filled with gel. Water flooding was then performed after gelation at 120 °C, and the pressure change was recorded.

**Figure 15:**
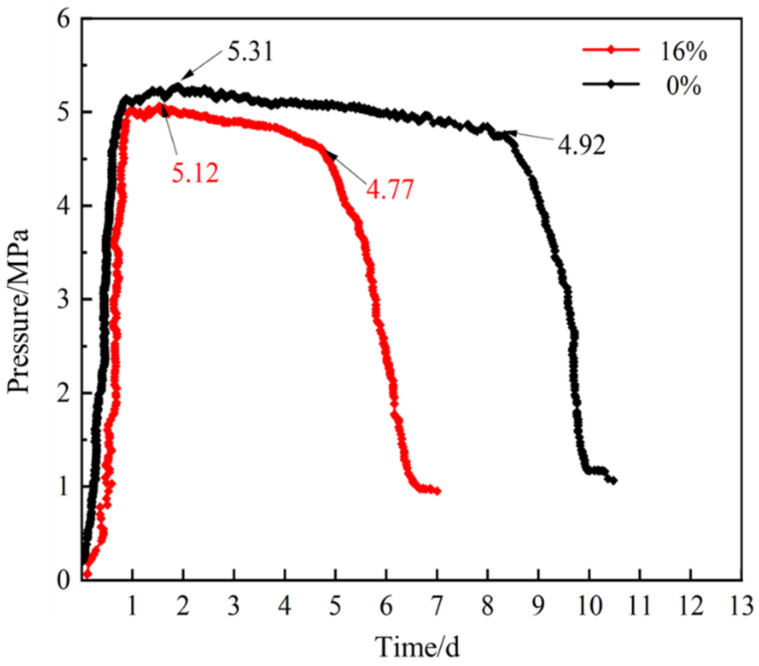
Breakthrough pressure in the wedge-shaped fracture core with a 3 mm outlet width.

**Figure 16:**
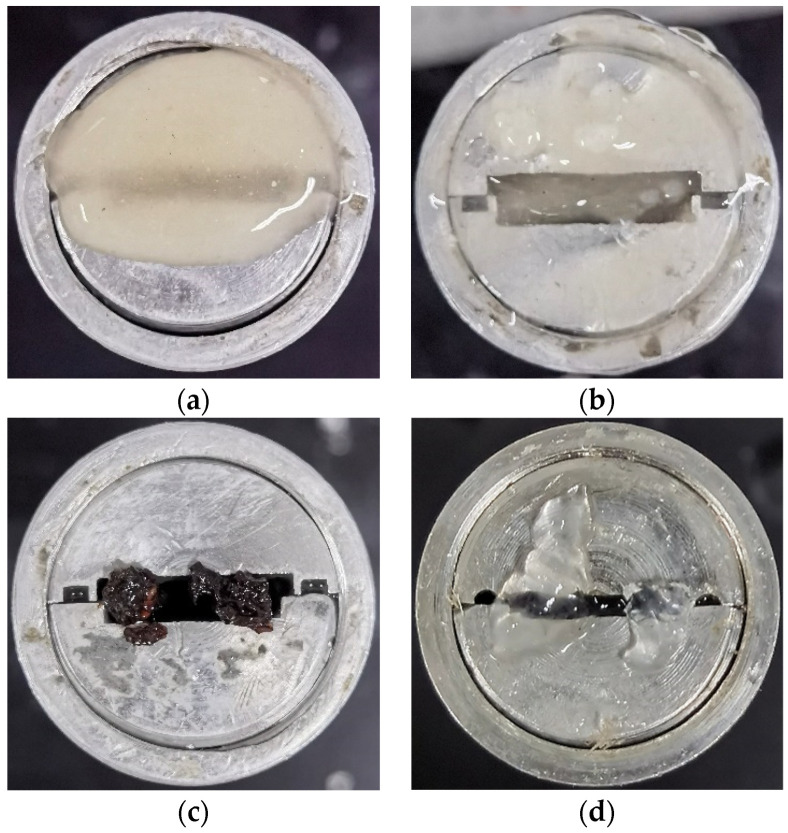
Outlet-end states of the 3 mm wedge-shaped fracture core under different conditions: (**a**) with capsule gel breaker; (**b**) without capsule gel breaker; (**c**) after gel breaking with capsule gel breaker; (**d**) after gel breaking without capsule gel breaker.

As shown in Figure 15, the maximum breakthrough pressure of the gel containing 16 wt.% capsules was 5.31 MPa. After the pressure was maintained for 5 d, it decreased to 4.77 MPa, and then a rapid pressure release occurred because a flow channel formed inside the wedge-shaped fracture core. Gel residues were observed at the outlet, as shown in Figure 16c. The gel without capsules also exhibited a maximum breakthrough pressure of 5.31 MPa. However, its pressure was maintained for 9 d before dropping abruptly at 4.92 MPa, and larger gel blocks remained at the outlet (Figure 16d). These results indicate that, in a wedge-shaped fracture, the gel containing the encapsulated breaker could still provide effective temporary plugging and exhibit good gel-breaking performance. For the same outlet dimension, the breakthrough pressure in the wedge-shaped fracture core was higher than that in the parallel fracture core.

**Figure 17:**
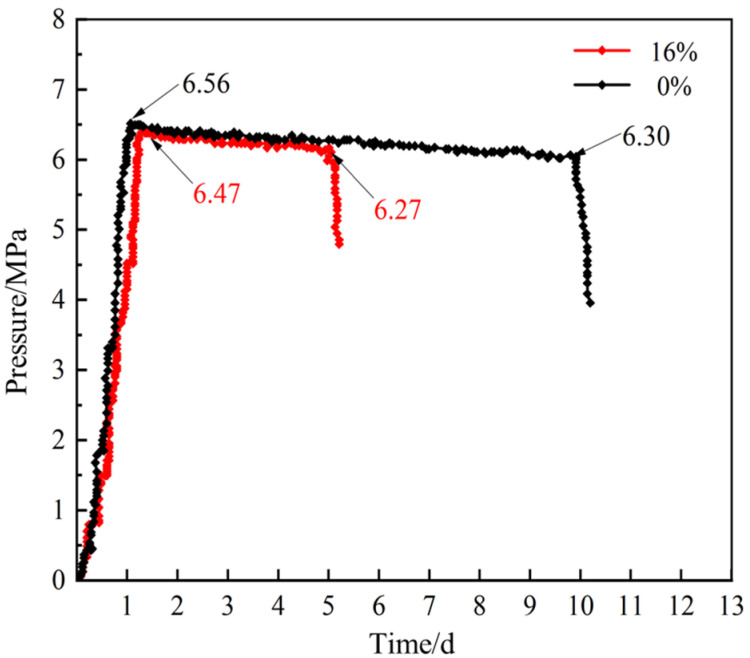
Breakthrough pressure in the packed-sand tube.

**Figure 18:**
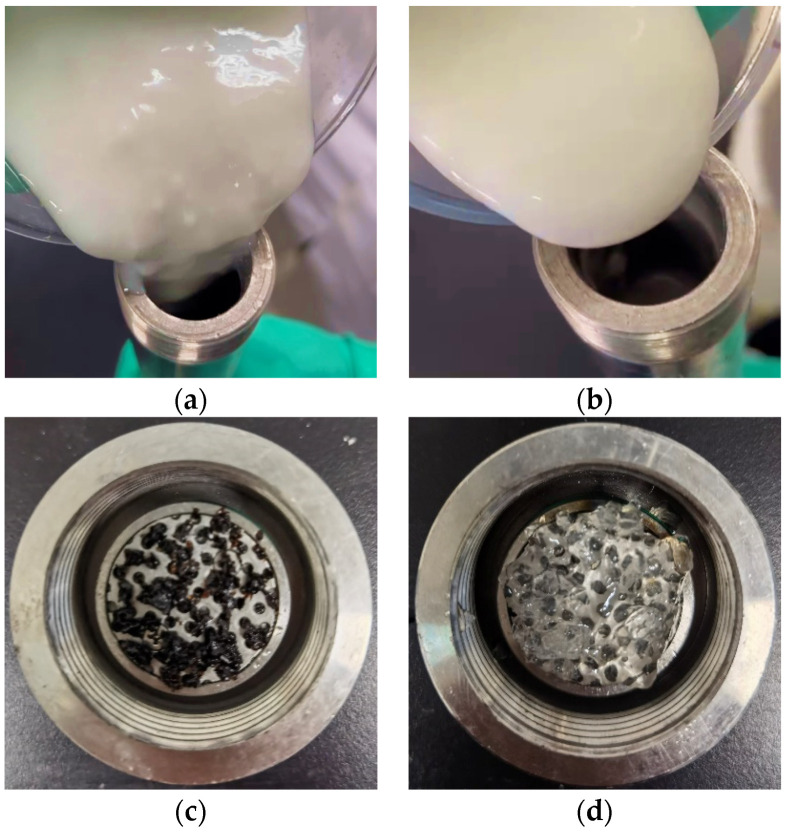
Gel injection and post-breaking states in the packed-sand tube: (**a**) with capsule gel breaker; (**b**) without capsule gel breaker; (**c**) after gel breaking with capsule gel breaker; (**d**) after gel breaking without capsule gel breaker.

Effect of the encapsulated breaker on gel temporary plugging in packed-sand tubes. A steel packed-sand tube with a length of 30 cm and an inner diameter of 5 cm was used to simulate an actual formation structure. The tube was filled with gravel, and one group was injected with gel containing 16 wt.% capsules, whereas the other group served as the blank control without capsules. After injection, the system was left undisturbed for a certain period, then connected to the pipeline, gelled at 120 °C, and subjected to displacement testing. In the packed-sand tube, the breakthrough pressure curves are shown in Figure 17. The maximum breakthrough pressure of the gel containing 16 wt.% capsules was 6.47 MPa. After the pressure had been maintained for 5 d, it declined to 6.27 MPa and then dropped rapidly. The outlet condition after gel breaking is shown in Figure 18c. For the gel without capsules, the maximum breakthrough pressure was 6.56 MPa. The pressure was maintained for 10 d before decreasing to 6.30 MPa and then falling rapidly, and the outlet condition is shown in Figure 18d. These results indicate that, whether or not the encapsulated breaker was added, the more intricate and finer flow channels in the packed-sand tube led to higher maximum breakthrough pressures and longer pressure-holding times than those observed in the parallel and wedge-shaped fracture cores.

### 2.4. Release and Gel-Breaking Mechanism of the UF-EC/SA Double-Shell Encapsulated Breaker

**Figure 19:**
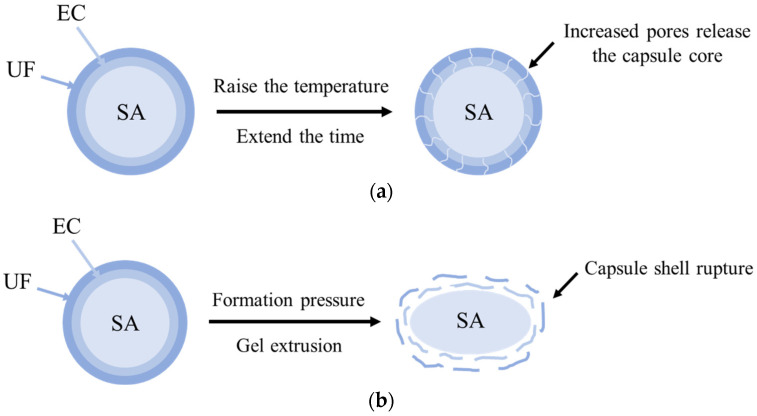
Schematic illustration of (**a**) permeation release and (**b**) extrusion-induced rupture release of the encapsulated breaker.

**Figure 20:**
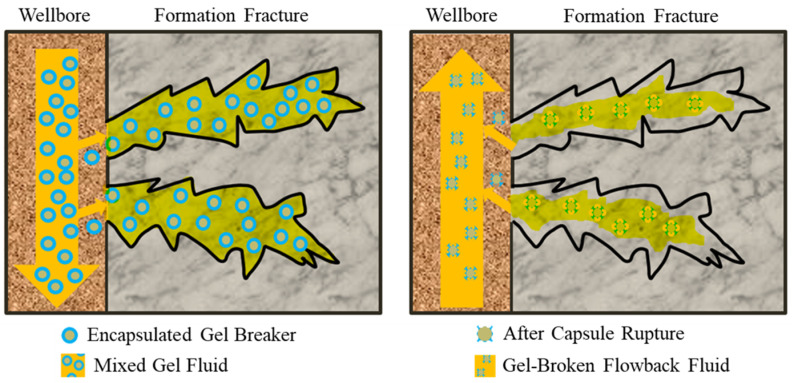
Schematic illustration of field application of the encapsulated breaker.

Under different temperatures and formation conditions, the encapsulated breaker can be released in different ways. Two major release pathways are involved: permeation release and extrusion-induced rupture release. Permeation release mechanism: Under ambient and dry conditions, the physicochemical properties of the capsules remain stable, allowing storage for a certain period. As shown schematically in Figure 19a, with increasing exposure time and/or increasing environmental temperature, the capsule shell softens and the pre-existing pores in the shell enlarge under heating, causing gradual release of the core material through the pores. A longer action time and a higher temperature both increase the release amount. In low-pressure and high-temperature formations, permeation release dominates, and the gel-breaking time can therefore be controlled by adjusting the capsule dosage (Figure 20). Extrusion release mechanism: When the capsules are mixed with the gel and injected into the formation, the capsule structure remains intact and does not release prematurely, thereby avoiding early viscosity loss of the gel. After gelation, the capsules are subjected to formation pressure and the force generated by compression of the gel. Once this force exceeds the pressure-bearing capacity of the capsule shell, the shell ruptures, as shown in Figure 19b, and the core material is rapidly released. In high-pressure formations, extrusion-induced rupture becomes the main release mode, although permeation release still coexists (Figure 20).

## 3. Conclusions

(1) Using oil-phase phase separation combined with in situ polymerization, a high-temperature-resistant UF-EC/SA double-shell encapsulated breaker was successfully prepared, in which UF resin served as the outer shell, EC as the inner shell, and sulfamic acid as the core. Under the optimized preparation conditions, a core-to-wall mass ratio of 10:3, a Tween-80/EC-SA capsule mass ratio of 1.5:1, a stirring speed of 500 r/min, and UF prepolymer preparation conditions of n (urea):n (formaldehyde) = 1:1.5, pH 8, and 80 °C for 30 min, the capsules achieved an encapsulation efficiency of 76.7%, had a uniform particle size of approximately 1.5 mm, exhibited a dense and smooth shell, and could withstand temperatures up to 150 °C, meeting the requirements for high-temperature formation operations.
(2) FTIR, TGA, XRD, and SEM characterization showed that the crystal structure of sulfamic acid remained intact in the UF-EC/SA capsules and that the physicochemical properties of the core material were unchanged after encapsulation. The double-shell structure was successfully formed. The capsules were stable in the range of 0–100 °C, while the shell gradually ruptured and released the core in the range of 120–140 °C. The outer UF shell significantly enhanced the overall thermal stability, and the capsules exhibited temperature-responsive sustained-release behavior, enabling controllable gel-breaking time.
(3) The UF-EC/SA encapsulated breaker exhibited excellent compatibility with the polymer gel and did not inhibit gelation within the temperature range of 80–150 °C or at dosages of 0–16 wt.%. Under highly mineralized conditions of 100,000 mg/L Na^+^ and 5000 mg/L Ca^2+^, the gel still maintained good mechanical properties, and the storage modulus remained higher than the loss modulus, indicating excellent salt tolerance.
(4) The gel-breaking time could be accurately regulated by the combined effects of temperature and capsule dosage; a higher temperature and a higher dosage both accelerated gel breaking. At 150 °C with 16 wt.% capsules, the gel was completely broken within 2.5 d. The residue concentration was only 351 mg/L, and the residue size was mainly concentrated within 100–500 μm, indicating thorough degradation and easy flowback, which helps avoid secondary reservoir damage.
(5) High-temperature/high-pressure displacement tests confirmed that the gel containing 16 wt.% capsules exhibited excellent temporary plugging performance in both fracture cores and packed-sand tubes. In the 3 mm wedge-shaped fracture core, the initial breakthrough pressure reached 5.31 MPa and remained stable for 5 d. In the packed-sand tube, the initial breakthrough pressure reached 6.47 MPa and was also maintained for 5 d. After gel breaking, a flow channel could be formed rapidly, showing excellent synergy among temporary plugging, gel breaking, and flowback. Therefore, the system is promising for plugging and channeling-control operations in high-temperature fractured formations and karst-fracture reservoirs.
(6) In the formation, the UF-EC/SA encapsulated breaker operates through the combined action of permeation release and extrusion-induced rupture release. In low-pressure/high-temperature environments, permeation-controlled sustained release dominates, whereas, in high-pressure formations, the shell is crushed under pressure, resulting in rapid gel breaking. The released sulfamic acid destroys the gel crosslinked network and polymer chains through H^+^-mediated reactions, thereby ensuring complete degradation of the gel and providing a new mechanism and material for efficient cleanup of temporary plugging gels in high-temperature oil and gas reservoirs.

## 4. Materials and Methods

### 4.1. Materials

Sulfamic acid (AR, 99.5%), urea (AR, 99.5%), polyethylene (AR, 99.5%), cyclohexane (AR, 99.5%), sodium chloride (AR, 99.5%), and calcium chloride (AR, 99.5%) were purchased from Shanghai Macklin Biochemical Co., Ltd. (Shanghai, China). Ethyl cellulose (CP), formaldehyde solution (ACS, 37 wt.%), acrylamide (AR, 99%), and N-hydroxymethylacrylamide (AR, 98%) were obtained from Shanghai Aladdin Reagent Co., Ltd. (Shanghai, China). Span-80 (AR, 99.5%), Span-60 (AR, 99.5%), Tween-80 (CP), Tween-60 (CP), and SDBS (95%, mixture) were purchased from Sinopharm Chemical Reagent Co., Ltd. (Shanghai, China). The high-temperature gel solution and deionized water were prepared in the laboratory.

### 4.2. Preparation of the UF-EC/SA Encapsulated Breaker

#### 4.2.1. Preparation of EC/SA Inner Capsules

Using oil-phase phase separation, cyclohexane was added to a three-neck flask and heated in a water bath. Ethyl cellulose and polyethylene were then added and stirred until completely dissolved. Sulfamic acid was subsequently introduced and, after particles appeared in the system, petroleum ether was added. The mixture was then cooled to room temperature, followed by filtration, washing, and drying to obtain EC/SA capsules.

#### 4.2.2. Preparation of UF Prepolymer

Urea and 35 wt.% formaldehyde solution were weighed according to a molar ratio of n (urea):n (formaldehyde) = 1:1.5 and dissolved under stirring in a beaker. The pH was adjusted to 8 using triethanolamine. The mixture was heated to 80 °C in a water bath and stirred at constant temperature for 30 min to obtain a slightly viscous, semi-transparent UF prepolymer, which was then cooled for later use.

#### 4.2.3. Preparation of UF-EC/SA Double-Shell Capsules

Tween-80 and EC/SA capsules were mixed at a mass ratio of 1.5:1 and stirred for 30 min to obtain a stable emulsion. The emulsion was transferred into a three-neck flask and heated in a 60 °C water bath under stirring at 500 r/min. The UF prepolymer was added dropwise, followed by five drops of n-octanol as a defoamer. A 0.1 g/mL ammonium chloride solution was then added dropwise to adjust the pH, and the reaction was allowed to continue for 2 h. After the reaction, the product was washed several times with deionized water and absolute ethanol and then dried at 60 °C to obtain the UF-EC/SA double-shell encapsulated breaker.

#### 4.2.4. Encapsulation Efficiency of UF-EC/SA Double-Shell Capsules

The encapsulation efficiency was determined using an extraction method. A certain mass of dried encapsulated-breaker sample (Wi) was weighed and washed twice with deionized water and ethanol to remove impurities. After centrifugal separation, the white lower-layer solid was collected, dried at 60 °C to constant weight, and weighed (Wr). The encapsulation efficiency was calculated according to Equation 1. Each experiment was repeated three times, and the average value was used.

Em = Wi/Wr × 100%,(1)

### 4.3. Preparation of the Polymer Gel

The polymer gel system used in this work was prepared from acrylamide (AM, AR, 99%) as the main monomer, N-hydroxymethylacrylamide (NHMA, AR, 98%) as the hydroxymethyl-functional comonomer/crosslinking component, crosslinker BWL, a rheology modifier, a catalyst, a water-soluble resin toughener, and silicate fibers. Crosslinker BWL was a hydroxymethyl-containing water-soluble resin-type crosslinker/toughener used in the laboratory formulation; its main reactive groups were hydroxymethyl (-CH2OH) groups, which can undergo dehydration condensation with amide groups on acrylamide-based polymer chains to form a three-dimensional network. The water-soluble resin toughener was used to increase the toughness and integrity of the gel network, and the silicate fiber was an inorganic chopped fiber added as a mechanical reinforcement phase. Based on optimization experiments, the best polymer-gel formulation was deter-mined as follows: polymer content, 10 wt.%; crosslinker BWL, 0.8 wt.%; rheology modifier, 2 wt.%; catalyst, 0.02 wt.%; water-soluble resin toughener, 2 wt.%; and silicate fiber, 1.5 wt.%.

### 4.4. Characterization of the UF-EC/SA Double-Shell Encapsulated Breaker

#### 4.4.1. FTIR Characterization of the UF-EC/SA Double-Shell Capsules

A Fourier transform infrared spectrometer (FTIR-7600, Shanghai Precision Scientific Instrument Co., Ltd., Shanghai, China) was used. Before testing, the instrument was calibrated for wavenumber and sensitivity using a KBr standard sample. The dried encapsulated breaker was mixed with KBr at a mass ratio of 1:99, fully ground, and pressed into pellets. The scanning range was 400–4000 cm^−1^, and 35 scans were collected to identify the functional groups in the capsules and analyze the interaction between the core and shell materials.

#### 4.4.2. Thermogravimetric Analysis of the UF-EC/SA Double-Shell Capsules

A TGA/DTA thermal analyzer (METTLER TOLEDO, Greifensee, Switzerland) was used. Uniformly sized samples of UF-EC/SA capsules, ethyl cellulose, and UF resin were separately placed in the sample chamber. Nitrogen was used as the protective atmosphere. The heating rate was set at 10 °C/min, and the temperature was increased from 30 °C to 600 °C. The change in sample mass with temperature was recorded to analyze the thermal stability and thermal degradation behavior of the capsules and their individual components.

#### 4.4.3. Micromorphology Characterization of the UF-EC/SA Double-Shell Capsules

A field-emission scanning electron microscope (Quanta 200F, FEI, Hillsboro, OR, USA) was used to observe the micromorphology of the encapsulated breaker and thereby evaluate shell roughness and surface features. The capsule samples were sputter-coated with gold to provide conductivity. The electron beam emitted by the SEM interacted with the sample surface, and the secondary-electron or backscattered-electron signals were converted into brightness or grayscale variations, yielding high-resolution images of the capsule surface.

#### 4.4.4. Powder X-Ray Diffraction of the UF-EC/SA Double-Shell Capsules

A powder X-ray diffractometer (Olympus, Tokyo, Japan) was used. Samples of UF-EC/SA capsules and pure sulfamic acid crystals were loaded evenly onto the sample stage. Wide-angle scans were carried out over a 2theta range of 5–90 degrees at a scanning rate of 10 degrees/min using a Cu target and a step size of 0.02. The diffraction data were processed with MDI Jade 6 software for peak fitting and phase analysis. The positions and intensities of the characteristic diffraction peaks were compared to verify the integrity of the sulfamic acid crystal structure after encapsulation.

### 4.5. Compatibility and Gel-Breaking Performance Evaluation of the UF-EC/SA Double-Shell Encapsulated Breaker in the Polymer Gel System

#### 4.5.1. Salt-Tolerance Evaluation

Gel solutions were prepared with brines containing different concentrations of Na^+^ (0, 3 × 10^4^, 5 × 10^4^, 7 × 10^4^, and 1 × 10^5^ mg/L) and Ca^2+^ (0, 500, 1500, 3000, and 5000 mg/L). After adding 8 wt.% capsules and completing gelation, the storage modulus and loss modulus were measured using the same rheometer conditions as described above to evaluate the effects of salt type and salinity on gelation performance.

#### 4.5.2. Effects of Temperature and Capsule Dosage on Gel-Breaking Performance

A multifactor coupling experiment involving temperature (80, 90, 100, 110, 120, 130, 140, and 150 °C) and capsule dosage (0%, 4%, 8%, 12%, and 16%) was designed. The gel-capsule systems with different formulations were placed in a roller heating furnace at the corresponding temperatures. The gel viscosity was measured every 12 h using a rheometer. According to the petroleum industry standard, a viscosity ≤5 mPa s was taken as complete gel breaking. Each experiment was repeated three times, and the average value was reported.

#### 4.5.3. Evaluation of Temporary Plugging Performance

A high-temperature/high-pressure plugging-displacement apparatus was used to simulate a 120 °C formation environment. Parallel fracture cores with outlet widths of 5 mm and 3 mm, a wedge-shaped fracture core with a 3 mm outlet width (all 30 cm in length), and a packed-sand tube of 30 cm × 5 cm were used as test media. The gel containing 16 wt.% capsules and a blank gel without capsules were injected separately at an appropriate pump rate. When gel appeared at the outlet, the injection valve was closed, the residual gel in the pipeline was removed, and the system was held at temperature for 10 h for gelation. Water flooding and pressurization were then performed. When the pressure began to fluctuate downward, pressurization was stopped and the maximum breakthrough pressure and the pressure-holding time were recorded. After gel breaking, the outlet residue and flowback behavior were observed to evaluate the temporary plugging effect and post-breaking cleanup performance.

## Figures and Tables

**Table 1:** Effect of core-to-wall mass ratio on the encapsulation efficiency of EC/SA capsules.

Mass Ratio	10:1	10:2	10:3	10:4
Encapsulation efficiency/%	63.4	74.6	85.3	85.6

**Table 2:** Effect of the UF prepolymer/EC/SA capsule mass ratio on the encapsulation efficiency of UF-EC/SA capsules.

Mass Ratio	10:1	10:3	10:5	10:7	10:9
Encapsulation efficiency/%	72.8	74.6	76.7	70.7	61.2

## Data Availability

The original contributions presented in this study are included in the article. Further inquiries can be directed to the corresponding author.
